# New observations in Central Italy of groundwater responses to the worldwide seismicity

**DOI:** 10.1038/s41598-020-74991-0

**Published:** 2020-10-20

**Authors:** Marino Domenico Barberio, Francesca Gori, Maurizio Barbieri, Andrea Billi, Antonio Caracausi, Gaetano De Luca, Stefania Franchini, Marco Petitta, Carlo Doglioni

**Affiliations:** 1 Earth Sciences Department, Sapienza University of Rome, P.le Aldo Moro 5, 00185 Rome, Italy; 2 Consiglio Nazionale Delle Ricerche, IGAG, Rome, Italy; 3 National Institute of Geophysics and Volcanology, Palermo, Italy; 4 National Institute of Geophysics and Volcanology, National Earthquake Observatory, L’Aquila, Italy; 5 National Institute of Geophysics and Volcanology, Rome, Italy

**Keywords:** Geophysics, Hydrogeology, Seismology, Tectonics

## Abstract

Chemical and physical responses of groundwater to seismicity have been documented for thousands of years. Among the waves produced by earthquakes, Rayleigh waves can spread to great distances and produce hydrogeological perturbations in response to their passage. In this work, the groundwater level, which was continuously recorded in a monitoring well in Central Italy between July 2014 and December 2019, exhibited evident responses to dynamic crustal stress. In detail, 18 sharp variations of the groundwater level due to worldwide M_w_ ≥ 6.5 earthquakes were observed. Apart from earthquakes that occurred in Papua New Guinea and those with a hypocentral depth > 150 km, all far away M_w_ ≥ 7.6 earthquakes produced impulsive oscillations of groundwater. As the earthquake magnitude decreased, only some earthquakes with 6.5 ≤ M_w_ < 7.6 caused groundwater level perturbations, depending on the data acquisition frequency and epicentral distance from the monitoring well. A clear correlation between earthquake distance and magnitude in hydrogeological responses was found. Our results shed light on the hydrosensitivity of the study site and on the characteristics of fractured aquifer systems. Detecting the water table variations induced by distant earthquakes is another step towards a correct identification of (preseismic) hydrogeological changes due to near-field seismicity.

## Introduction

**Figure 1:**
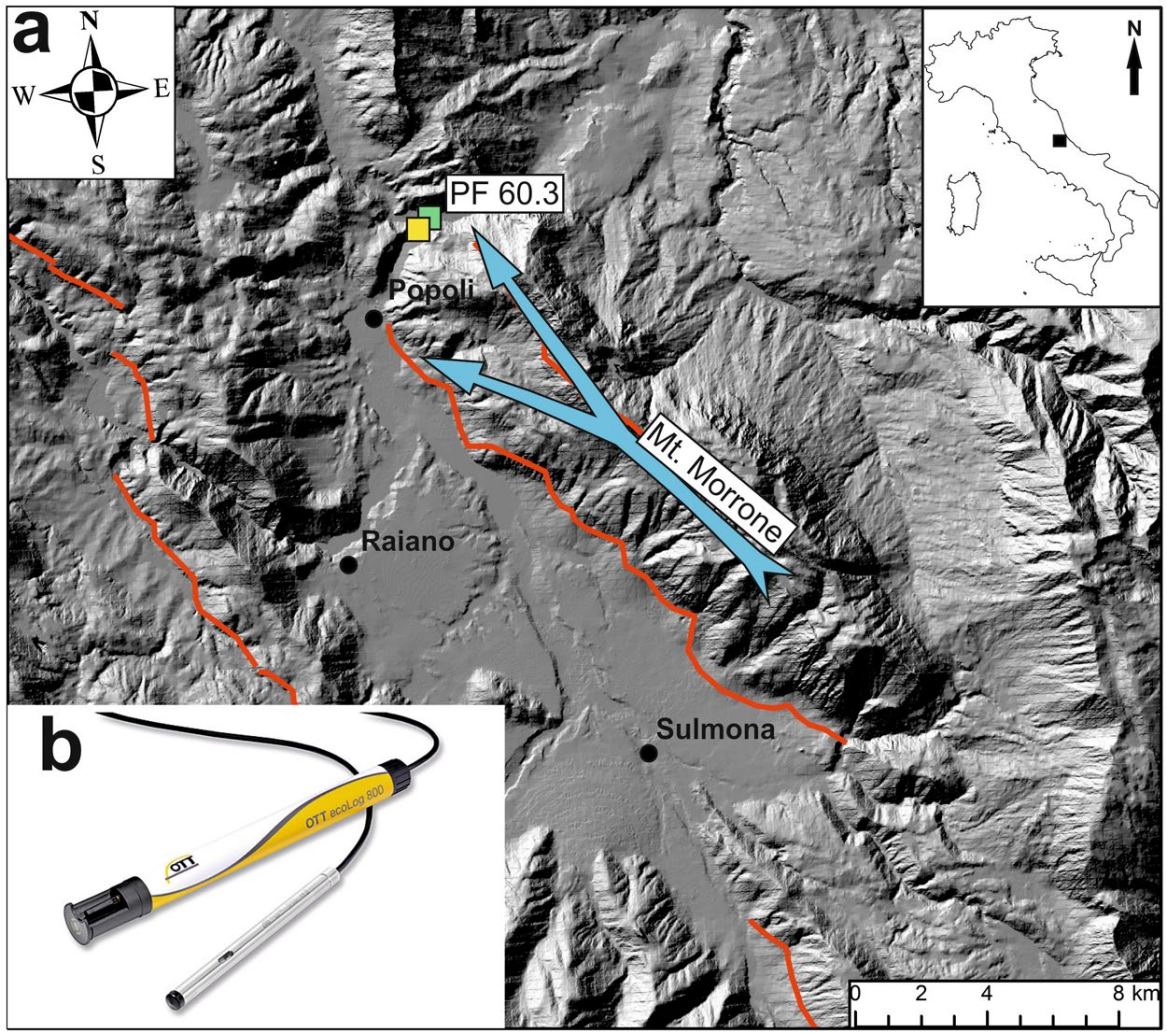
Study area. This figure has been drawn using CorelDRAW^32^. (**a**) Map of Central Apennines (see location in upper right inset). Active faults (all extensional) are from the Ithaca database (available online at https://www.isprambiente.gov.it/en/projects/soil-and-territory/italy-hazards-from-capable-faulting). Base digital elevation model is from the ISPRA database SINAnet (available online at https://www.sinanet.isprambiente.it/it). Locations of the PF60.2 and PF60.3 wells monitored in this work are displayed with yellow and green symbols, respectively. The blue arrow indicates the principal groundwater flow path of the Mt. Morrone aquifer; (**b**) OTT ecoLog800 multiparametric probe for continuous acquisition and remote transmission of data, including groundwater level, temperature, and electrical conductivity.

Earthquakes are among the main natural processes that can cause the strongest perturbations in the Earth’s crust. Seismic events can change crustal stress, both static and dynamic, in the co-seismic and post-seismic phases. Static stress is generated by fault loading and its release mostly occurs in the near-field whereas dynamic stress is triggered by fault slip, generating the migration of seismic waves and, for this reason, its perturbation also spreads to the far-field. Many studies have highlighted the sensitivity of fluid behaviour related to the modification of the stress field, both the static and the dynamic one^1,2^. In particular, hydrogeological and geochemical responses include: changes in groundwater level^3–5^, temperature^6,7^, water chemistry^8–10^, stream flow^11–13^, and gas geochemistry^14–18^. Among these parameters, a change in groundwater level is more commonly recorded due to the simplicity of doing so through the use of inexpensive devices. In fractured high-permeability aquifers, like the one studied in this work, impulsive changes in the water table are frequent, also due to classical hydrogeological causes. Various mechanisms have been proposed to explain groundwater level responses to earthquakes including: (1) poroelastic response to co-seismic static strain^19^; (2) undrained consolidation of sediments^20^; (3) clogging–unclogging of pores and fractures by oscillatory flows produced by the passage of seismic waves^3,21,22^; (4) co-seismic gas bubble development^23,24^; (5) shaking-induced compaction or dilatation^25^. Depending on the mechanism involved, the temporal pattern of groundwater level variation can vary significantly and, hence, can be symptomatic of specific mechanisms. Previous studies have highlighted permanent and transient signals that are characterized by step-like and/or spike-like temporal changes both upward and/or downward^26–28^. Only a few studies have reported groundwater level variations induced by earthquakes that are very far away from the observation point, known as ‘teleseism’^3,25,26,29,30^. According to the USGS a teleseism is a tremor related to seismic events occurring more than 1000 km away^31^. To investigate the relationship between groundwater characteristics and the seismic cycle, two multiparameter probes were installed in a 50 m and a 100 m deep groundwater wells (PF60.2 and PF60.3) in the Central Apennines (Italy, Fig. 1, see “Material and methods” section) in 2014.

These wells were part of a monitoring test site in fractured carbonate aquifers, developed for the identification of potential hydrogeochemical precursors of earthquakes^5,9^. After their installation (July 2014 for PF60.3 and November 2014 for PF60.2) the probe in the PF60.3 well recorded significant changes in groundwater level following the occurrence of many high magnitude earthquakes around the world (M_w_ ≥ 6.5). In this paper, we present the results of the water level monitoring with a view to expanding the understanding of perturbations of fluids in the upper crust due to earthquakes, with a focus on groundwater–earthquake physical relationships. Since these or similar probes are used by us and other geoscientists to identify potential hydrogeochemical precursors to nearby earthquakes^2,5,9,20,33,34^, identifying and thus filtering the effect on (and also the cause of) the groundwater level induced by distant earthquakes is a pre-requisite to understanding the possible effect induced by the seismic cycle of nearby faults.

## Geological and hydrogeological settings

The Central Apennines fold-and-thrust belt was formed by tectonic accretion and the following backarc extension during the Oligocene-Quaternary ‘westward’ subduction and ‘eastward’ retreat of the Adriatic plate beneath the European plate. This segment of mountain chain is characterized by NE-verging thrusts, which dissect the tectonic edifice into several thick tectonic sheets^35^. The post-accretion extensional regime, which has dominated the Central Apennines from the Pliocene onwards, has segmented the orogenic edifice and determined the current structural setting. The development, in particular, of NW-striking extensional faults has been responsible of intra-mountain basin formation (e.g. the Campo Imperatore, L’Aquila, Fucino, and Sulmona basins). These basins are mostly half-grabens filled by Upper Pliocene and Pleistocene continental deposits. At present, the extensional regime is particularly seismogenic along the axis of the Central Apennines, while the compressional regime is active along the eastern margin of the Apennines and the Western Adriatic Sea. The monitoring wells are located in the hanging wall of the Mt. Morrone normal fault system (Fig. 1), where the current rates of extension measured by the GPS network are 3–4 mm/years^36^. The Apennines belt is characterized by huge fractured aquifers hosted by Meso-Cenozoic carbonate sequences forming the Apennines thrust sheets. The aquifer systems, characterized by high transmissivity and huge flow through springs with a stable and huge discharge, are often separated and sealed by low-permeability layers (aquicludes), such as siliciclastic marine and continental deposits. The study site, in particular, is located between the Gran Sasso and the Mt. Morrone carbonate aquifers (Fig. 1). Groundwater flow feeds base-flow springs in the gorges of the Pescara River^9^ [references therein].

## Results

**Figure 2:**
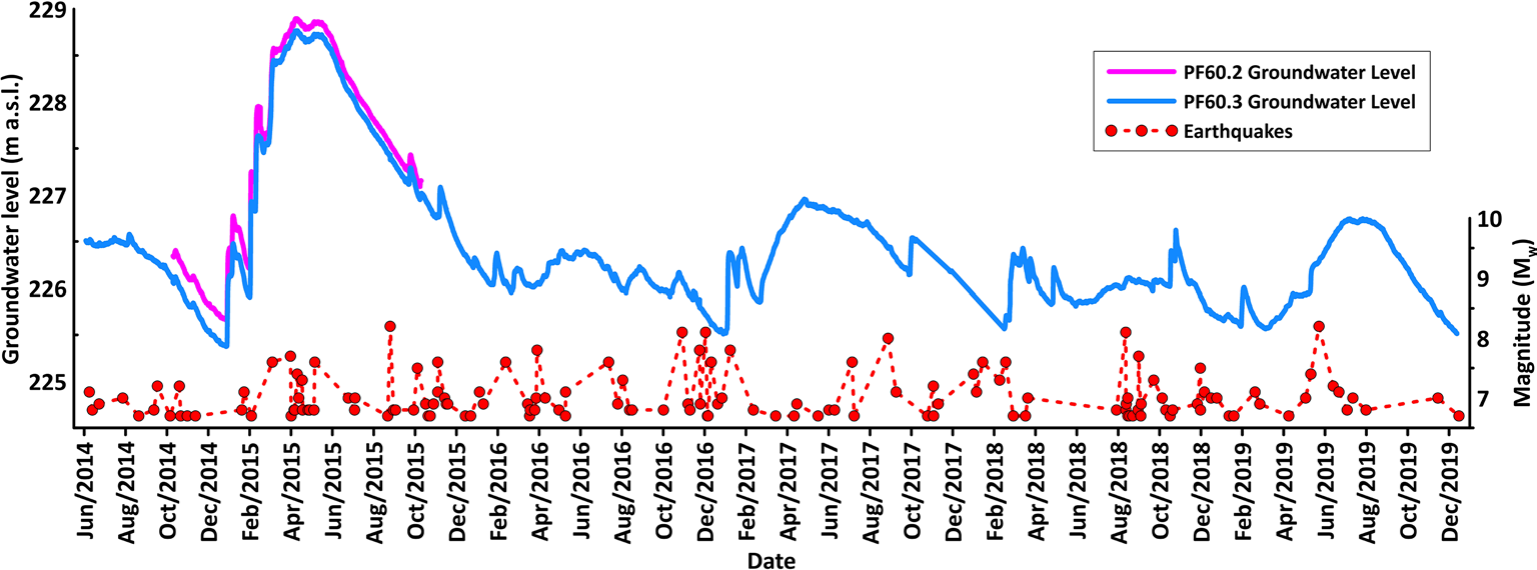
Time series. This figure has been realized using Grapher 7. (**a**) Time series (July 1st, 2014–December 31st, 2019) of groundwater level recorded in the PF60.3 well (100 m deep; Fig. 1a) is shown with the blue line; time series (October 1st, 2014–November 30th, 2015) of groundwater level recorded in the PF60.2 well (50 m deep; Fig. 1a) is shown with the pink line. (**b**) Time series (July 1st, 2014–December 31st, 2019) of magnitude (M_w_ ≥ 6.5) for earthquakes occurred worldwide in the same period of the hydrogeological monitoring is shown with red dots. Seismic data are from the INGV database (available online at https://terremoti.ingv.it/).

**Figure 3:**
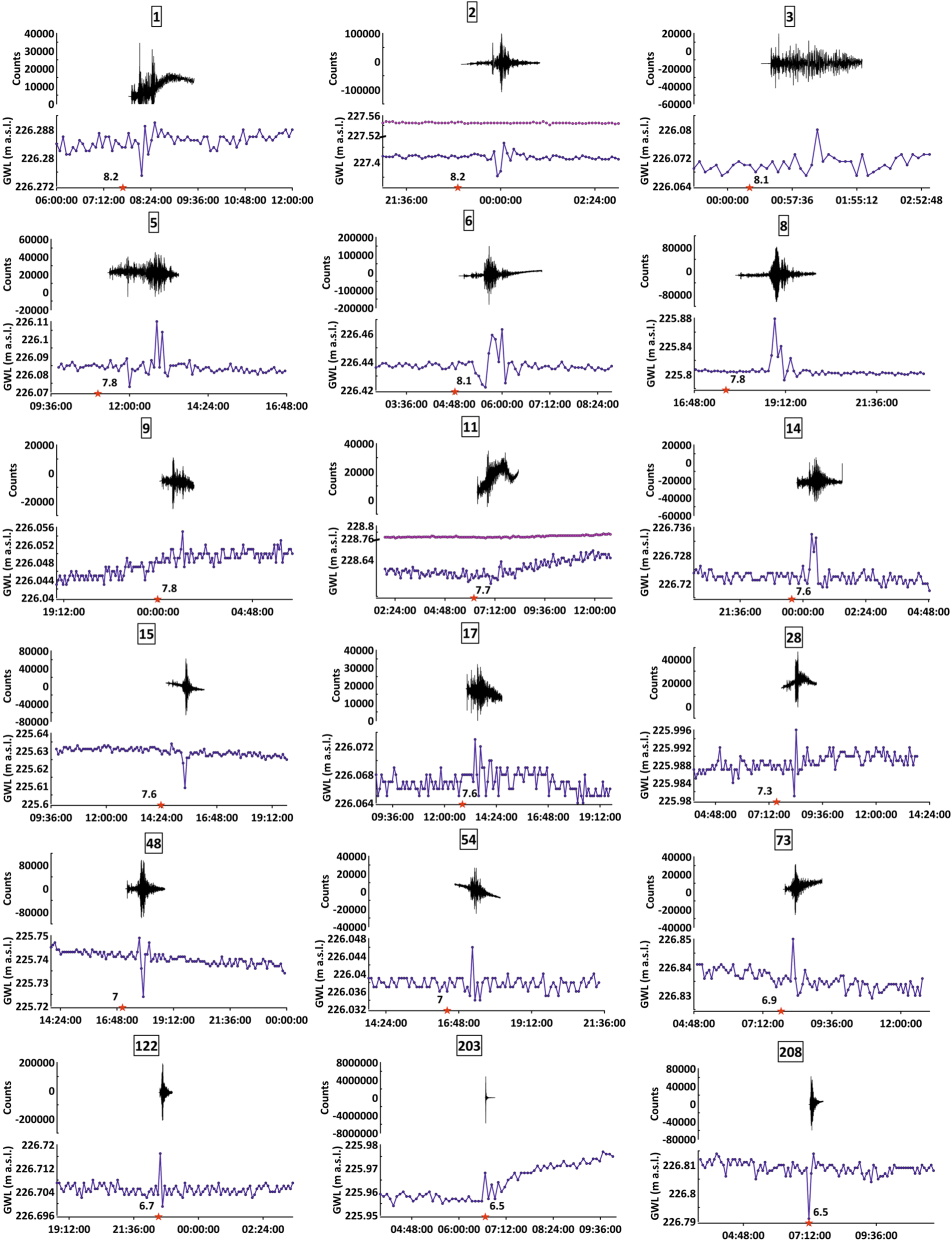
Groundwater level, seismic trace, and earthquakes. This figure has been realized using Grapher 7. 18 cases of interaction between signals are exhibited. The groundwater level is shown with the blue line (recorded in PF60.3) and the pink line (recorded in PF60.2), the vertical component of ground motion from local seismic stations (T0110 and INTRodacqua) is displayed with the black line and seismic events are shown with red stars. On the Y axis of the seismic trace, counts are directly proportional to the ground velocity. Seismic data are from the INGV database (available online at https://terremoti.ingv.it/). The numbers on the top of each diagram refer to the earthquake ID reported in the Supplementary Table 1.

**Figure 4:**
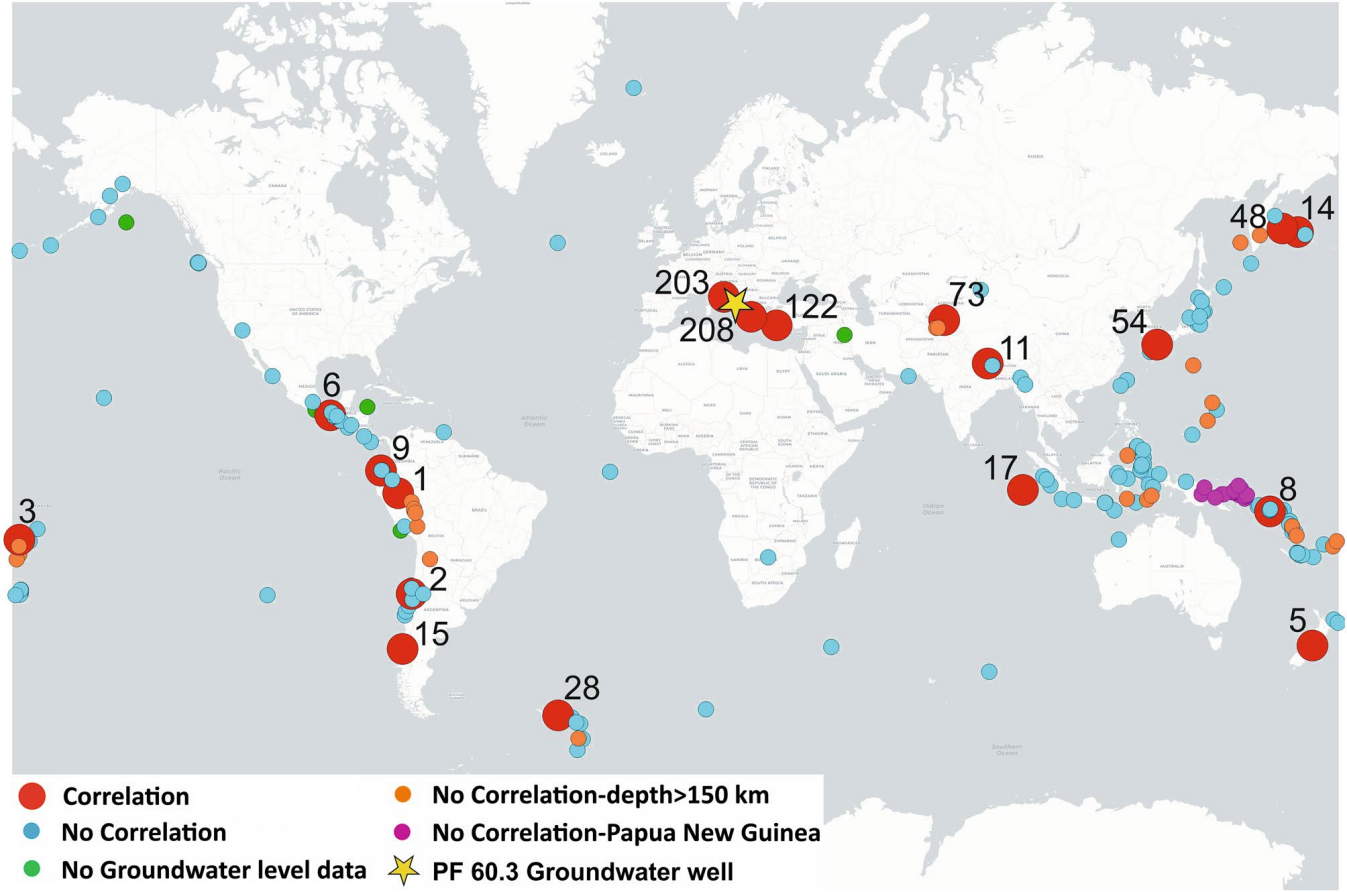
The worldwide distribution of earthquakes (M_w_ ≥ 6.5). This figure has been realized using QGIS 3.6^37^. The 218 seismic events are displayed with circles (the different colours refer to the classification of Fig. 5 in the “Discussion” section) and are reported with the respective number of the Supplementary Table 1. Seismic data are from the INGV database (available online at https://terremoti.ingv.it/).

**Figure 5:**
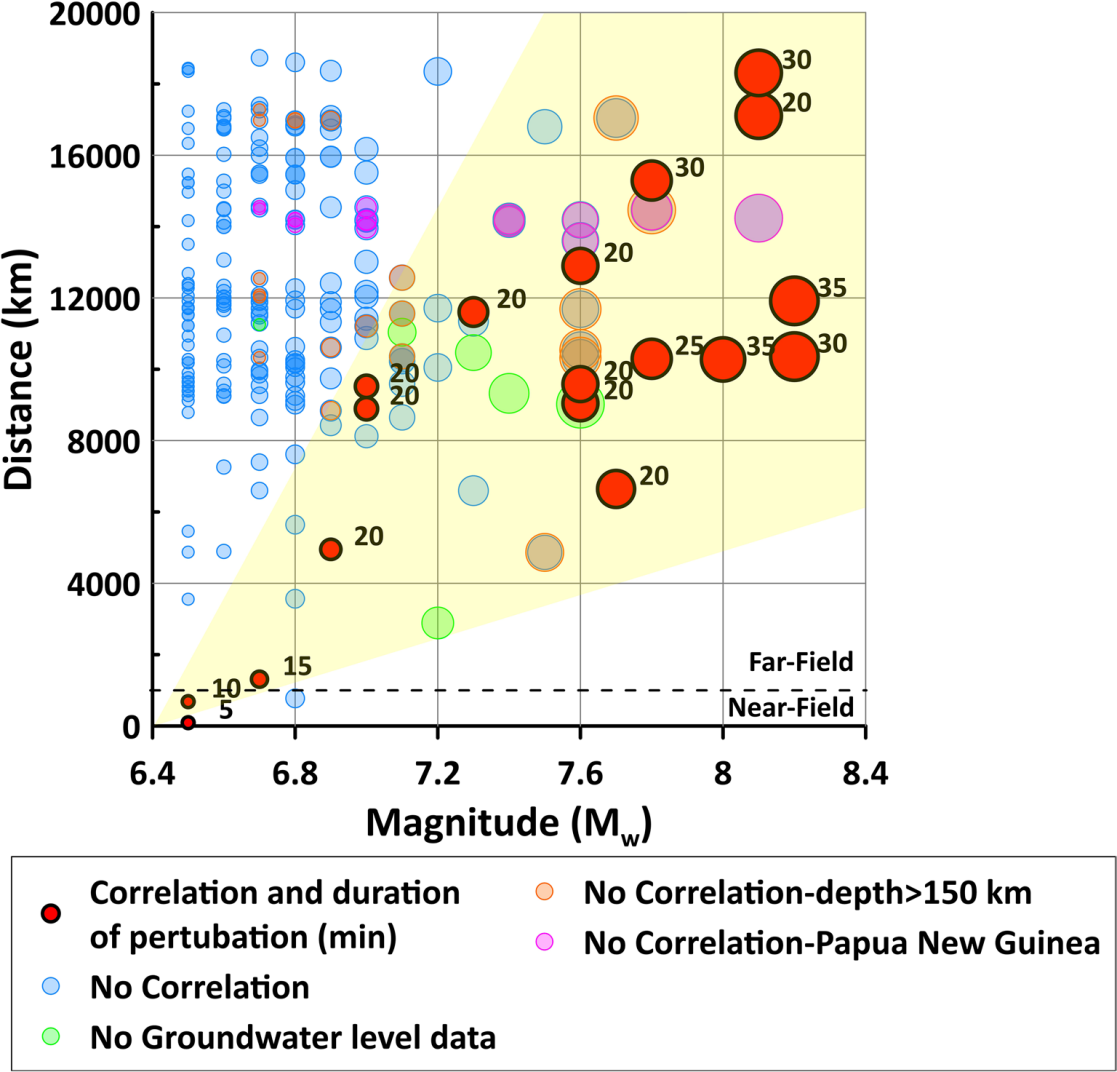
Distance from wells vs earthquake magnitude. This figure has been realized using Grapher 7. On the Y axis, epicentral distance from the monitoring wells (PF60.2 and PF60.3) is shown; on the X axis, the magnitude of the seismic events (M_w_ ≥ 6.5) occurred in the same period of our hydrogeological monitoring is shown. Seismic data are from the INGV database (available online at https://terremoti.ingv.it/). 18 earthquakes, for which a correlation with groundwater level changes has been identified, are displayed with red circles of different sizes depending on the magnitude. A number is also reported indicating the duration of perturbation. The remaining earthquakes are displayed with blue, green, orange, and pink circles and did not cause appreciable variation in the groundwater level. Correlation between magnitude and distance is displayed with the yellow box.

The groundwater level data were continuously recorded (every five minutes) from July 2014 to December 2019 in PF60.3 and from November 2014 to October 2015 in PF60.2 (Fig. 2). The acquired data highlight that the water table is characterized by a typical seasonal variation of about 1.5 m and a maximum oscillation of about 3.40 m (i.e. the change between minimum and maximum values, February 2015–June 2015), which is a very limited variation in agreement with the base-flow conditions in the observed discharge area (Fig. 2). No active withdrawals influencing the groundwater level are ongoing in this aquifer, as documented by previous monitoring^9^. This monitoring also showed that local rainfall has a negligible effect on the groundwater level due to the location in the discharge zone of the aquifer^9^. In Fig. 2, we show the earthquakes with M_w_ ≥ 6.5 that occurred worldwide in the same period of hydrogeological monitoring. Detailed analyses of groundwater level data corresponding to all 218 seismic events were processed, and 18 characteristic groundwater level behaviours were found and are shown in Fig. 3 together with the seismic trace of the vertical component recorded by two nearby seismometers (the number on the top of each diagram corresponds to the earthquake ID in the Supplementary Table 1). All the 18 cases selected are characterized by anomalous groundwater level changes recorded only in the PF60.3 well within one hour, at most, from the occurrence of the seismic event, depending on the epicentral distance. Moreover, these variations are described by upward, downward or both spike changes because of the data acquisition moment of the probe (every five minutes) in relation to the arrival of Rayleigh waves. Hence, the gap between two consecutive recordings implies that the acquisition of groundwater level data can occur at any time of the seismic wave passage. In addition, other trends and smaller spikes (e.g. in diagrams 11 and 203) can be attributed to further hydrological perturbations, such as the recharge and discharge of the aquifer. However, the main impulsive peaks induced by distant earthquakes are clearly discernible and related to the ground motion as also shown by the vertical component of the seismic trace. When the counts (directly proportional to the velocity of ground motion) were less of about 20,000, the groundwater level exhibited less evident responses (this condition was found for example in diagrams 3 and 9 in Fig. 3). The maximum amplitude and the duration of the groundwater level perturbation range between the maxima of 8.7 cm and 35 min, respectively, and the minima of 0.8 cm and 5 min, respectively (see Supplementary Table 1). The worldwide distribution of earthquakes (with M_w_ ≥ 6.5) is shown in Fig. 4, where the abovementioned 18 seismic events are displayed with red circles (other colours in Fig. 4 refer to the classification of Fig. 5 in the “Discussion” section).

## Discussion

The results suggest that, in the aquifer where the PF60.3 well is located, the passage of seismic waves due to the strongest earthquakes worldwide (M_w_ ≥ 6.5) produced abrupt fluctuations in groundwater level in the Central Apennines. No other classical hydrological process has indeed involved the study area (e.g. local and anthropic perturbation or near-field earthquakes). Two simultaneous mechanisms can be recognized as the main cause for the observed fluctuations: (1) rapid pressure changes in the aquifer as the body rock is dilated and compressed by earthquake waves passing through it, and (2) amplification by the water column momentum moving in the well, due to the fractured system^30,38–40^. Previous works^30,38^ proposed that groundwater level fluctuations in a well can be induced only by those waves that produce rock body volume changes, the so-called ‘dilatational waves. Both P waves and the later arriving Rayleigh waves own this property, with the latter being expected to generate the larger dilatational oscillations owing to their long periods (typically ~ 20 s, with wavelength ~ 100 km)^41–44^. On the contrary, S and Love waves, which produce shear motion as they propagate through rocks, are not expected to produce significant volume changes. Moreover, the groundwater response to P waves cannot be determined by our monitoring system due to a measurement frequency (once every five minutes) that is not high enough to accurately record P waves, which have frequencies of several cycles per second^38,40^. Hence, it is commonly assumed that, in the far-field (> 1000 km), water level responds only to long-period surface Rayleigh waves^3,30,39,45^. Based on these previous studies, we interpreted the recorded groundwater level changes as driven by the passage of remotely-generated Rayleigh waves (teleseisms) through the aquifer.

In our temporal series (Fig. 2), we identified 18 main events where the groundwater level in the PF60.3 well exhibited an anomalous spike change both/either upward and/or downward (Figs. 3 and 4 red circles). Given the above consideration about the acquisition gap of five minutes, the shape and the amplitude of the perturbation did not reflect the real waveform. Thus, the physical insignificance of the recorded upward and/or downward spikes did not allow us to group these effects into different classes. To understand the interaction between groundwater level and far-field seismicity, the relationships between earthquake magnitude, distance from the well, and hypocentral depth were analysed (Fig. 5). We observed a significant response to all the strongest seismic events in the world with a M_w_ ≥ 7.6 (Fig. 5 red circles), except for those that occurred in Papua New Guinea (Fig. 5 pink circles) and for those occurring at depths greater than 150 km (Fig. 5 orange circles). The hypocentral depths of the 18 events under consideration reached a maximum of 150 km, except one that was deeper (depth 574 km for the M_w_ 8.1 of Fiji Islands on August 19, 2018 see Supplementary Table 1). Among the correlations observed, 11 events were characterized by M_w_ between 7.6 and 8.2, whereas the other seven seismic events had an earthquake M_w_ between 6.5 and 7.5. Thus, as the distance between the monitoring well and the earthquake epicentre decreases, a relationship was observed even for earthquake magnitudes less than 7.6 (Fig. 5 smaller red circles). Among these seismic events that induced groundwater responses, only two were located within the near-field seismicity (Figs. 3 and 5). For example, the 2016 M_w_ 6.5 Norcia earthquake is the sole case within the near-field where a step change was observed, with a water table increase lasting for several days after the earthquake^5,9,22,46^. However, a correlation between epicentral distance from the wells and earthquake magnitude is evident (Fig. 5 yellow box), confirming that these two parameters are the two most relevant factors in controlling the responses of groundwater level at a specific site^6,47^. It is not surprising that earthquakes with magnitudes close to our lower limit (M_w_ = 6.5), occurring at great distances, did not have an effect on the groundwater level data. The remaining seismic events did not cause appreciable variation in the water table (Fig. 5, blue circles) and six of them occurred in periods of a gap in groundwater level monitoring due to probe maintenance work (Fig. 5, green circles). The relationship observed between the epicentral distance and the duration of perturbation on groundwater level data is indeed directly proportional (Fig. 5, black numbers). Moreover, during the period of hydrogeological monitoring, transient changes induced by the passage of seismic waves did not produce any variation in chemical parameters (temperature and electrical conductivity) at the PF60.3 well, in addition to the piezometric level. Indeed, the perturbations derived from teleseismic waves are not able to produce a rock–water–gas interaction strong enough to consequently cause hydrogeochemical changes (e.g. variations in chemical-physical parameters, gas content, and/or mixing between deep and shallow fluids), because the static stress does not involve the aquifer, which simply hosts the seismic waves propagation from far seismic events. The absence of any geochemical variations confirms that fluids moving within the well and the surrounding rocks have an impulse that is negligible to the kinetic of reaction (e.g. water–rock).

Furthermore, a significant exception of non-relationship between groundwater level and far-seismicity is represented by strong earthquakes in Papua New Guinea (Fig. 5, pink circles). It is worth noticing that an ‘anomalous’ absence of groundwater level variation corresponding to this seismicity was found for all the seismic events that occurred in Papua New Guinea, about 14,000 km away from the monitoring wells. Tectonically, Papua New Guinea is located at the triple junction of the Eurasian, Indo-Australian, and Pacific plates. It presents some of the most complex tectonic activity, including oceanic crust subduction and arc continent collision^48^. A wide Bouguer gravity anomaly is caused by the uneven density distribution in the crust and upper mantle. Therefore, this gravitational anomaly and a surrounding larger anelasticity of the lithosphere can provide a comprehensive explanation for this unrecorded event^49^. The variation in the crustal structure within Papua New Guinea could cause the dispersion of Rayleigh waves^50^ affecting their potential perturbation at a great distance, as in our monitoring site. This hypothesis was supported by ground velocity data from two considered local seismometers (e.g. counts less of about 20,000).

We also observed a few cases of no manifest interaction occurring between the groundwater level and earthquakes even with a magnitude larger than 7.0 and regardless of the epicentral distance from the monitored site. A plausible explanation could be connected with low ground velocity variations induced by these seismic events and/or with the monitoring frequency of the probe (5 mins), which may have been insufficient to detect some high-frequency signals. This latter aspect would apply to those seismic events that occurred close to the monitoring well and were consequently characterized by short travel-time intervals of possibly lower than five minutes.

In order to assess the significance of the observed peaks owing to the arrival of Rayleigh seismic waves, where it was possible, groundwater level data were processed. In detail, groundwater time series, from a minimum of 3 to a maximum of 15 days around the seismic events (depending on the time series suitability) were detrended. Furthermore, mean values and the ± 2σ thresholds of groundwater level were calculated. Anomalous signals (e.g. groundwater level peaks recognized as results of interaction with seismic events) exceeding the ± 2σ confidence interval due to the occurrence of 14 out of the 18 earthquakes were pointed out (See Supplementary Table 2).

Another recent study^29^ conducted in China has corroborated our results and strengthened the relationship between teleseismic waves and groundwater level responses. Despite only having one monitored well, these authors observed 61 responses from distant earthquakes. In their study, in contrast to our work, the sampling frequency rate is 1 sample/s. For this reason, they detected more interactions and identified also nearby earthquakes with a faster perturbation and a lower magnitude. However, it is noteworthy that 10 out of our 18 identified interactions were also recorded by the monitoring well in China.

We also analysed possible relationships between the following parameters: the amplitude of groundwater level perturbation vs. earthquake magnitude and the duration of groundwater level perturbation vs. earthquake magnitude. As already discussed, a five-minute gap between every groundwater level acquisition implies that the real maximum amplitude of oscillations could be bigger than that recorded. Consequently, the expected relationship between the amplitude of groundwater level perturbation vs. earthquake magnitude has not been found. On the contrary, the relationship between the duration of groundwater level perturbation and earthquake magnitude follows a direct linear correlation, described by a Pearson’s coefficient r = 0.83 with p value < 0.05. Thus, the relationship has been statistically verified. As observed, the strongest earthquakes produced the longest perturbations. At the same time the hypocentral depth influenced the seismic wave propagation. Indeed, the M_w_ 8.1 Fiji Islands earthquake with a hypocentral depth of 574 km produced a shorter perturbation (20 min) than other earthquakes of a similar magnitude (see Supplementary Fig. 1 and Supplementary Table 1).

The results also highlighted the aspect named “Hydrosensitivity” of the monitoring site^5,9,51,52^. With the term hydrosensitivity we refer to the ability of a hydrogeological system to respond to external perturbations (e.g. discharge and recharge period, earthquakes, tides). Other hydrogeological monitoring stations in Central Italy, equipped with the same instrumentation and the same sampling frequency, have not so far recorded any correlation between groundwater level variation and far-field seismicity. For instance, we have previously mentioned that in the November 2014 to October 2015 period the groundwater level response to seismicity recorded in the 100 m deep well PF60.3 did not match with that recorded in the 50 m deep well PF60.2, which was only 3 m away from the PF60.3. The main difference between these two wells is their depth and the lithology of the drilled rock bodies: the PF60.3 well is instated into the fractured carbonate bedrock hosting the Mt. Morrone aquifer, whereas the PF60.2 well is instated into the alluvial deposits. Furthermore, other monitoring sites also located in fractured aquifers in Central Italy did not show such water table oscillations. Consequently, the possibility of observing groundwater level response to far-field seismicity is strongly related to the selection of the monitoring site, and should take into consideration several criteria^52^, the first of which is the difference between porous and fractured aquifers, where the latter can clearly show responses to teleseismic activity.

Other similar research carried out by international teams in Iceland, Japan, China, and Korea^19,34,53,54^ has been aimed at identifying potential changes in groundwater level and in chemical content due to the occurrence of earthquakes. Unlike our work, these studies were not specifically devoted to far-field seismic events. They focused on fluid behaviour during the seismic cycle, which is mainly defined by the groundwater level decreasing prior to the earthquake in connection with fracture opening and permeability change^54^. For example, three phases of groundwater level were recorded since 230 days before the M_w_ 7.6 Chi-Chi earthquake in Taiwan. These variations were characterized by a sharp and significant decrease (phase 1), a subsequent rise (phase 2), and a stop of groundwater level uplift about 13 days prior to the earthquake (phase 3)^53^. Furthermore, in southern Iceland, coseismic and post-seismic water level changes in geothermal wells located in a seismic zone were detected, following two M_w_ 6.5 earthquakes^19^. Finally, in the Central Apennines (Central Italy), significant hydraulic pressure and electrical conductivity anomalies were identified five days before the M_w_ 6.0 Amatrice earthquake^46^. Apart from these trends, the hydraulic pressure data showed coseismic effects related to the M_w_ 8.2 Chile earthquake (on September 16th, 2015) where the arrival of long-period Rayleigh waves was very clear^55^. As shown in our study (Fig. 3), the typical impulsive peak-like response of groundwater level to the passage of Rayleigh waves in the monitoring site is easily discernible because of its characteristics, which differ from other perturbations (e.g. the effects of rainfall, the recharge and discharge of the aquifer, the effects of near-field seismicity). In particular, this work allowed us to recognize these transient perturbations and exclude them as a potential signal caused by near-field seismicity. Knowing about and discerning the effect of all the parameters (including teleseism) that can influence the hydrogeological and hydrogeochemical features is a fundamental pre-requisite for the investigation of the potential effects of nearby earthquakes.

## Conclusions

- The PF60.3 monitoring well, equipped with a multiparametric probe OTT ecoLog800 in a fractured carbonate aquifer, allowed us to identify groundwater level fluctuations related to the passage of Rayleigh seismic waves deriving from distant earthquakes.
- Owing to their impulsive character, the oscillations induced by teleseismic waves are clearly recognisable with respect to classical water table changes correlated with the hydrogeological cycle and/or human induced changes.
- The peculiar responses of the monitoring well PF60.3 highlighted once again the potential sensitivity of the fractured aquifers to the strain variation in pre-selected hydrogeological conditions.
- Since many groups in seismic regions of the world are now running monitoring stations to identify potential hydrogeological precursors to large near-field earthquakes (e.g. Italy, China, Iceland, Japan, and Korea), the characterization and identification of teleseismic effects on these monitoring stations are a pre-requisite for the correct recognition of near-field seismic effects. This recognition may be due to the typically impulsive peak-like response of the groundwater level to strong teleseisms, which is markedly different from that driven by near-field earthquakes.

## Material and methods

In this study, we used both hydrogeological and seismic data to identify interactions between groundwater levels and distant earthquakes. The hydrogeological monitoring data are from the PF60.2 and PF60.3 wells, which are 50 m and 100 m deep boreholes drilled through alluvial deposits and the Mt. Morrone regional carbonate aquifer, respectively (Fig. 1). The PF60.2 and the PF60.3, which are located at Lat. 42.196875°, Long. 13.852316°, altitude 238.90 m, and Lat. 42.196910°, Long. 13.852372°, altitude 238.95 m, respectively, were equipped with multiparametric probes (OTT ecoLog800) to measure the groundwater levels in 2014. These probes are equipped by an automatic system for continuous data acquisition and remote data transmission, concerning groundwater level, temperature, and electrical conductivity (groundwater level: resolution 0.001 m, error ± 0.05%; temperature: resolution 0.001 °C, error 0.1 °C; electrical conductivity: resolution 0.001 mS/cm, error ± 0.5%). The sample frequency of data measurement was set to five minutes. The barometric pressure of groundwater level measurements was automatically compensated for. With respect to the seismic data, we used the database run by the National Seismic Network (from the website https://terremoti.ingv.it/). Data were filtered to select the main seismic events (n. 218 events with M_w_ ≥ 6.5, see Supplementary Table 1), which occurred worldwide in the same period of our hydrogeological monitoring at the PF60.2 and PF60.3 wells (July 2014–December 2019, Fig. 2b). Supplementary Table 1 summarises the main parameters for every recorded seismic event (i.e. time (UTC), latitude, longitude, depth (km), magnitude (M_w_), event location name, the occurrence of groundwater level change, epicentral distances from the monitoring wells, maximum amplitude (cm) and duration (min) of perturbation on groundwater level). For the 18 seismic events that caused groundwater level responses, data from two local seismic stations were also considered. Specifically, the T0110 seismic station, located about 10 km from the PF60.3 well, was used. This station belongs to the National Seismic Network of the Centro Nazionale Terremoti of the Istituto Nazionale di Geofisica e Vulcanologia (CNT-INGV) and is equipped with a broadband Trillium 120 s compact seismometer (see online at https://terremoti.ingv.it/instruments/station/T0110). Only during the occurrence of two teleseismic events (n. 48 and 122) the T0110 seismic station was out of order. Hence, for these two latter cases, the reported data are from another station named INTRodacqua, which is located about 20 km from our monitoring site and it is equipped with a Trillium 40 s seismometer (see online at https://terremoti.ingv.it/instruments/station/INTR). For the elaboration, only the vertical seismic component of ground motion was used.

## Supplementary information

Supplementary Information.
