# Supplementary material for: New observations in Central Italy of groundwater responses to the worldwide seismicity
Source: Sci Rep. 2020 Oct 20;10:17850. doi: 10.1038/s41598-020-74991-0 (PMC7575607; doi:10.1038/s41598-020-74991-0)
Supplement: Supplementary file 1 — Supplementary Information. [file 41598_2020_74991_MOESM1_ESM.pdf]

First observation in central Italy of groundwater responses to the worldwide seismicity

Marino Domenico Barberio[1,*], Francesca Gori[1], Maurizio Barbieri[1], Andrea Billi[2], Antonio Caracausi[3], Gaetano De Luca[4], Stefania Franchini[1], Marco Petitta[1], Carlo Doglioni[1,5]

(1) Earth Sciences Department, Sapienza University of Rome, Rome, Italy,

(2) Consiglio Nazionale delle Ricerche, IGAG, Rome, Italy

(3) National Institute of Geophysics and Volcanology, Palermo, Italy

(4) National Institute of Geophysics and Volcanology, National Earthquake Observatory, L'Aquila, Italy

(5) National Institute of Geophysics and Volcanology, Rome, Italy

(*) Corresponding author: Marino Domenico Barberio, Earth Sciences Department, Sapienza University of Rome, P.le Aldo Moro 5, 00185, Rome, Italy; email: marinodomenico.barberio@uniroma1.it tel. (+39) 3200443022

Supplementary Figure 1

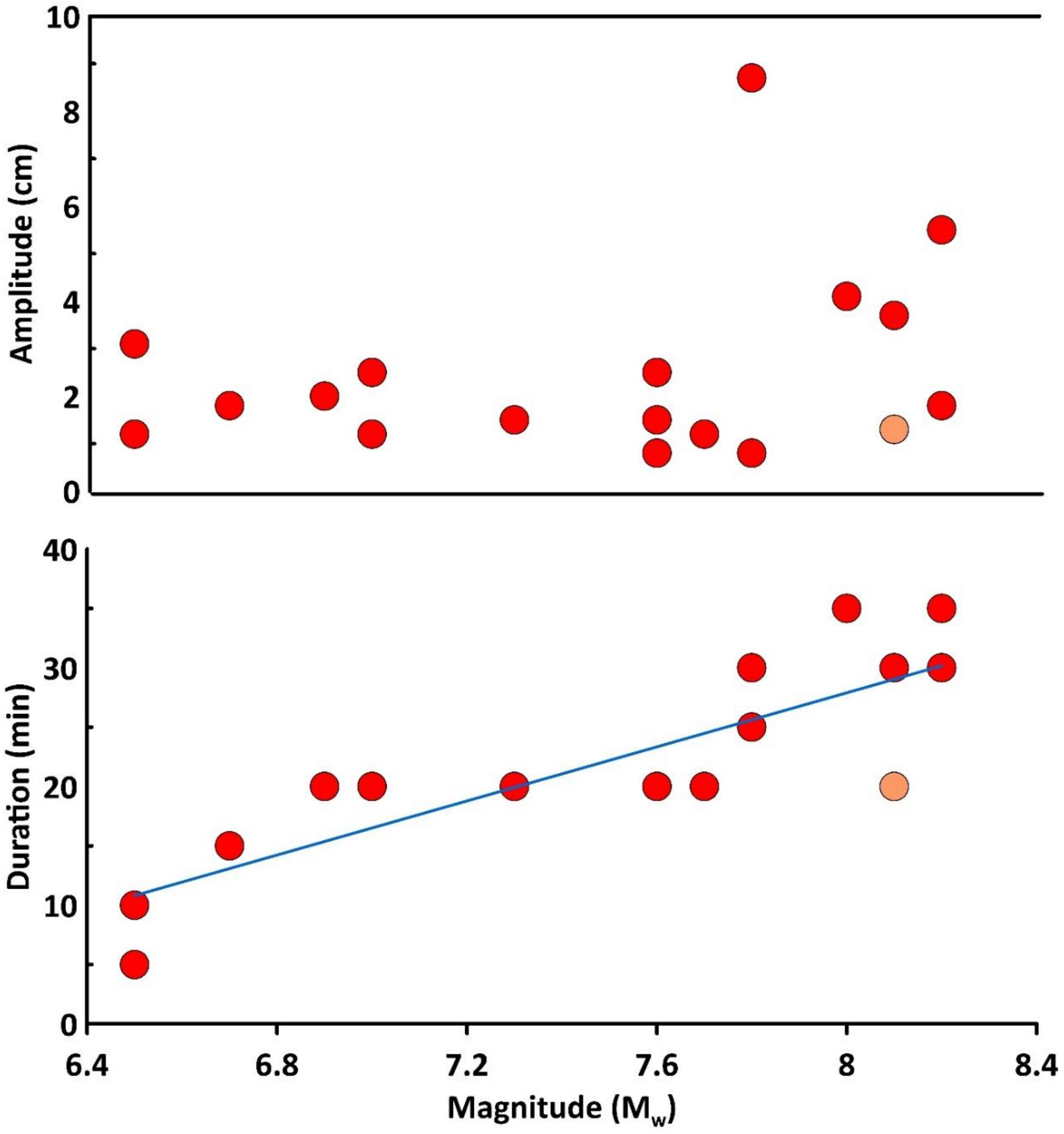

Supplementary Figure 1: Amplitude and duration of groundwater level perturbation vs earthquake magnitude for 18 earthquakes selected in Fig. 5 (for which a correlation with groundwater level changes has been identified). This figure has been realized using Grapher 7. (a) Earthquake magnitude vs amplitude of groundwater level perturbation relationship. (b) Earthquake magnitude vs duration of groundwater level perturbation relationship. Seismic events with a hypocentral depth within 150 kilometres are displayed with red circles; the only case of deeper earthquake is displayed with an orange circle (depth: 574 km; M_w 8.1 in Fiji Islands region on August 19, 2018).

Supplementary Table 1

Earthquake ID	Time (UTC)	Latitude	Longitude	Depth (km)	Magnitude	EventLocationName	Changes	Distance (km)	Amplitude (cm)	Duration (min)
1	26/05/2019 07.41	-5.75859	-75.2555	133.6	8.2	Northern Peru [Land: Peru]	Correlation	10356	1.8	30
2	16/09/2015 22.54	-31.4	-71.6	20	8.2	Near coast of central Chile [Land: Chile]	Correlation	11919	5.5	35
3	19/08/2018 00.19	-18.0773	-178.066	574.2	8.1	Fiji Islands region [Sea: Fiji]	Correlation	17110	1.3	20
4	17/12/2016 10.51	-4.52109	153.471	108.6	8.1	Papua New Guinea [Sea]	No Correlation Papua New Guinea	14237		
5	13/11/2016 11.03	-42.5742	172.861	10.2	8.1	South Island, New Zealand [Land: New Zealand]	Correlation	18310	3.7	30
6	08/09/2017 04.49	15.1277	-93.6877	71.5	8	Near coast of Chiapas, Mexico [Sea: Mexico]	Correlation	10276	4.1	35
7	22/01/2017 04.30	-6.20859	155.074	164.8	7.8	Papua New Guinea [Land]	No Correlation Papua New Guinea	14467		
8	08/12/2016 17.38	-10.6418	161.343	34.8	7.8	Solomon Is. [Sea]	Correlation	15291	8.7	30
9	16/04/2016 23.58	0.371	-79.94	19.2	7.8	Near coast of Ecuador [Land: Ecuador]	Correlation	10294	0.8	25
10	06/09/2018 15.49	-18.4711	179.442	647.7	7.7	Fiji Islands [Sea: Fiji]	No Correlation depth>150km	17044		
11	25/04/2015 06.11	28.18	84.72	10	7.7	Nepal	Correlation	6645	1.2	20
12	25/02/2018 17.44	-6.03984	142.77	19.5	7.6	New Guinea, Papua New Guinea [Land: Papua New Guinea]	No Correlation Papua New Guinea	13603		

13	23/01/2018 09.31	55.9055	-149.049	10.2	7.6	United States [Sea]	No Groundwater level data	9020		
14	17/07/2017 23.34	54.4148	168.919	10.2	7.6	Russia [Sea]	Correlation	9046	1.5	20
15	25/12/2016 14.22	-43.2492	-74.0723	19.5	7.6	Southern Chile [Land: Chile]	Correlation	12900	2.5	20
16	29/07/2016 21.18	18.4148	145.662	164.8	7.6	Northern Mariana Islands-Guam [Sea]	No Correlation depth>150km	11685		
17	02/03/2016 12.49	-4.84805	94.2715	17.6	7.6	Southwest of Sumatera, Indonesia [Sea]	Correlation	9585	0.8	20
18	24/11/2015 22.50	-9.96328	-70.9383	624.2	7.6	Peru [Land]	No Correlation depth>150km	10324		
19	30/05/2015 11.23	27.7348	140.509	675.4	7.6	Japan [Sea]	No Correlation depth>150km	10538		
20	29/03/2015 23.48	-4.732	152.515	19.5	7.6	Papua New Guinea [Sea]	No Correlation Papua New Guinea	14187		
21	05/12/2018 04.18	-21.9445	169.293	19.5	7.5	New Caledonia [Sea]	No Correlation	16800		
22	26/10/2015 09.09	36.3586	70.7607	193	7.5	Afghanistan [Land]	No Correlation depth>150km	4867		
23	14/05/2019 12.58	-4.09922	152.613	19.5	7.4	Papua New Guinea [Sea]	No Correlation Papua New Guinea	14145		
24	10/01/2018 02.51	17.318	-83.621	10.2	7.4	North of Honduras [Sea: Honduras]	No Groundwater level data	9326		
25	05/05/2015 01.44	-5.44922	151.826	10.2	7.4	Papua New Guinea [Land]	No Correlation Papua New Guinea	14210		
26	28/09/2018 10.02	-0.260156	119.904	19.5	7.3	Minahassa Peninsula, Sulawesi, Indonesia [Land: Indonesia]	No Correlation	11348		

27	16/02/2018 23.39	16.5586	-97.7271	19.5	7.3	Oaxaca, Mexico [Land: Mexico]	No Groundwater level data	10468		
28	19/08/2016 07.32	-54.9914	-31.8359	19.5	7.3	South Georgia & the South Sandwich Is. [Sea]	Correlation	11605	1.5	20
29	12/05/2015 07.05	27.7102	86.1248	10.2	7.3	Nepal	No Correlation	6598		
30	15/06/2019 22.55	-30.4734	-177.747	67.2	7.2	Kermadec Islands, New Zealand [Sea: New Zealand]	No Correlation	18350		
31	12/11/2017 18.18	34.7977	45.8701	19.5	7.2	Iran [Land]	No Groundwater level data	2889		
32	15/11/2014 02.31	1.92	126.5	52	7.2	Indonesia [Sea]	No Correlation	11715		
33	14/10/2014 03.51	12.568	-88.026	92.6	7.2	Nicaragua [Sea]	No Correlation	10051		
34	24/06/2019 02.53	-6.38262	129.169	226.4	7.1	Indonesia [Sea]	No Correlation depth>150km	12568		
35	22/02/2019 10.17	-2.13047	-76.8867	141.4	7.1	Ecuador [Land]	No Correlation	10231		
36	11/12/2018 02.26	-58.4227	-26.3346	164.8	7.1	S. Geo. and S. Sandw. Is. [Land]	No Correlation depth>150km	11561		
37	14/01/2018 09.18	-15.7148	-74.5825	19.5	7.1	Peru (Peruvian point of view) [Sea]	No Groundwater level data	11040		
38	19/09/2017 18.14	18.6082	-98.426	72.3	7.1	Central Mexico [Land: Mexico]	No Correlation	10348		
39	28/05/2016 09.47	-56.0742	-26.9401	96.1	7.1	South Georgia & the South Sandwich Is. [Sea]	No Correlation	11563		
40	24/01/2016 10.30	59.727	-153.496	104.3	7.1	Southern Alaska, United States [Land: United States]	No Correlation	8651		
41	24/11/2015 22.45	-10.4836	-70.882	596.1	7.1	Peru [Land]	No Correlation depth>150km	10361		

42	16/02/2015 23.06	39.764	143.03	13.5	7.1	Off east coast of Honshu, Japan [Sea: Japan]	No Correlation	9596		
43	07/07/2014 11.23	14.8	-92.6	76	7.1	Near coast of Chiapas, Mexico [Sea: Mexico]	No Correlation	10226		
44	14/11/2019 16.17	1.56445	126.39	59	7	Indonesia [Sea]	No Correlation	11209		
45	14/07/2019 09.10	-0.643359	128.021	9.8	7	Halmahera, Indonesia [Land: Indonesia]	No Correlation	12030		
46	06/05/2019 21.19	-6.93633	146.464	139.5	7	Papua New Guinea [Land]	No Correlation Papua New Guinea	13956		
47	29/12/2018 03.39	5.86055	126.889	83.2	7	Indonesia [Sea]	No Correlation	11419		
48	20/12/2018 17.01	54.9633	164.701	10.2	7	Russia [Sea]	Correlation	8894	2.5	20
49	10/10/2018 20.48	-5.50547	151.137	19.5	7	Papua New Guinea [Land]	No Correlation Papua New Guinea	14167		
50	21/08/2018 21.31	10.6664	-62.8945	105.5	7	Near coast of Venezuela [Land: Venezuela]	No Correlation	8124		
51	29/03/2018 21.25	-5.49141	151.418	19.5	7	Papua New Guinea [Land]	No Correlation Papua New Guinea	14183		
52	10/01/2017 06.13	4.45078	122.66	632	7	Philippines [Sea]	No Correlation depth>150km	11208		
53	28/04/2016 19.33	-16.0699	167.421	12.1	7	Vanuatu Islands [Sea: Vanuatu]	No Correlation	16175		
54	15/04/2016 16.25	32.6057	130.713	10.4	7	Kyushu, Japan [Land: Japan]	Correlation	9522	1.2	20
55	04/12/2015 22.24	-47.5945	85.1331	19.5	7	Southeast Indian Ridge [Sea]	No Correlation	12169		
56	27/07/2015 21.41	-2.62617	138.526	18.4	7	Irian Jaya, Indonesia [Land: Indonesia]	No Correlation	13012		

57	18/07/2015 02.27	-10.3289	165.073	10.2	7	Solomon Is. [Sea]	No Correlation	15514		
58	07/05/2015 07.10	-7.22109	154.484	19.5	7	Papua New Guinea [Sea]	No Correlation Papua New Guinea	14538		
59	24/08/2014 23.21	-14.64	-73.53	60	7	Central Peru [Land: Peru]	No Correlation	10877		
60	01/03/2019 08.50	-14.618	-70.1001	258.6	6.9	Central Peru [Land: Peru]	No Correlation depth>150km	10610		
61	30/11/2018 17.29	61.3336	-150.05	19.5	6.9	Southern Alaska, United States [Land: United States]	No Correlation	8432		
62	10/09/2018 04.19	-31.6758	-179.097	135.2	6.9	New Zealand [Sea]	No Correlation	18368		
63	19/08/2018 14.56	-8.36367	116.722	9.8	6.9	Indonesia [Land]	No Correlation	11687		
64	19/11/2017 22.43	-21.3539	168.727	19.5	6.9	New Caledonia [Sea]	No Correlation	16720		
65	28/04/2017 20.23	5.50547	125.149	19.5	6.9	Philippines [Sea]	No Correlation	11327		
66	03/01/2017 21.52	-19.2937	175.904	18.8	6.9	South of Fiji Islands [Sea: Fiji]	No Correlation	16971		
67	09/12/2016 19.10	-10.9055	160.964	10.2	6.9	Solomon Is. [Sea]	No Correlation	15954		
68	21/11/2016 20.59	37.2955	141.642	10	6.9	Near east coast of eastern Honshu, Japan [Sea: Japan]	No Correlation	9745		
69	12/08/2016 01.26	-22.507	173.068	19.5	6.9	New Caledonia [Sea]	No Correlation	17100		
70	03/04/2016 08.23	-14.2102	166.765	19.5	6.9	Vanuatu Islands [Sea: Vanuatu]	No Correlation	15963		
71	30/01/2016 03.25	53.9051	158.593	152.7	6.9	Near east coast of Kamchatka Peninsula, Russia [Land: Russia]	No Correlation depth>150km	8840		

72	09/12/2015 10.21	-4.1168	129.533	34	6.9	Indonesia [Sea]	No Correlation	12414		
73	07/12/2015 07.50	38.032	72.9229	0.8	6.9	Tajikistan	Correlation	4952	2	20
74	18/11/2015 18.31	-8.99297	158.407	19.5	6.9	Solomon Is. [Sea]	No Correlation	14543		
75	07/11/2015 07.31	-30.7898	-71.4391	19.5	6.9	Near coast of central Chile [Land: Chile]	No Correlation	11883		
76	21/07/2014 14.54	-19.78	178.441	616.1	6.9	South of Fiji Islands [Sea: Fiji]	No Correlation depth>150km	16974		
77	02/08/2019 12.03	-7.30547	104.815	19.5	6.8	Southwest of Sumatera, Indonesia [Sea: Indonesia]	No Correlation	10633		
78	06/07/2019 03.19	35.8102	-117.536	19.5	6.8	Central California, United States [Land: United States]	No Correlation	10060		
79	05/12/2018 06.43	-22.057	169.614	19.5	6.8	New Caledonia [Sea]	No Correlation	16844		
80	25/10/2018 22.54	37.4924	20.595	10	6.8	Costa Occidentale Peloponneso (GRECIA)	No Correlation	774		
81	16/10/2018 01.03	-21.7055	169.522	10.2	6.8	New Caledonia [Sea]	No Correlation	16803		
82	05/09/2018 18.08	42.6586	141.885	19.5	6.8	Japan [Land]	No Correlation	9255		
83	19/08/2018 04.28	-16.95	-178.03	423	6.8	Fiji Islands region [Sea: Fiji]	No Correlation depth>150km	16983		
84	05/08/2018 11.46	-8.30039	116.497	9.8	6.8	Indonesia [Land]	No Correlation	11700		
85	22/06/2017 12.31	13.7004	-90.9045	73.8	6.8	Near coast of Guatemala [Sea: Guatemala]	No Correlation	10175		
86	14/06/2017 07.29	14.95	-91.93	125	6.8	Guatemala	No Correlation	10160		
87	24/02/2017 17.28	-23.0801	-178.804	434.8	6.8	South of Fiji Islands [Sea: Fiji]	No Correlation depth>150km	16975		

88	24/11/2016 18.43	11.918	-88.9087	19.5	6.8	El Salvador [Sea]	No Correlation	10176		
89	17/10/2016 06.15	-6.01172	148.816	63.3	6.8	Papua New Guinea [Sea]	No Correlation Papua New Guinea	14037		
90	01/09/2016 16.38	-37.1602	178.655	10.2	6.8	Off east coast of North Island, New Zealand [Sea: New Zealand]	No Correlation	18605		
91	29/08/2016 04.29	0.0632813	-17.7539	0.8	6.8	North of Ascension Island [Sea]	No Correlation	5643		
92	18/05/2016 16.46	0.485156	-79.6289	19.5	6.8	Near coast of Ecuador [Land: Ecuador]	No Correlation	10259		
93	13/04/2016 13.55	23.0273	94.8684	130.5	6.8	Myanmar [Land]	No Correlation	7616		
94	07/04/2016 03.32	-13.89	166.57	19.5	6.8	Vanuatu Islands [Sea: Vanuatu]	No Correlation	15923		
95	20/10/2015 21.52	-14.8148	167.322	135.2	6.8	Vanuatu Islands [Sea: Vanuatu]	No Correlation	15930		
96	24/09/2015 15.53	-0.57	131.26	18	6.8	Indonesia [Sea]	No Correlation	12276		
97	21/09/2015 17.39	-31.5633	-71.4551	19.5	6.8	Near coast of central Chile [Land: Chile]	No Correlation	11933		
98	27/07/2015 04.49	52.249	-169.53	15	6.8	Fox Islands, Aleutian Islands, United States [Sea: United States]	No Correlation	9112		
99	29/05/2015 07.00	56.7035	-156.673	34	6.8	Alaska Peninsula, United States [Sea: United States]	No Correlation	9009		
100	22/05/2015 23.59	-11.1023	163.162	10.2	6.8	Solomon Is. [Sea]	No Correlation	15458		
101	22/05/2015 21.45	-11.0602	163.557	0.8	6.8	Solomon Is. [Sea]	No Correlation	15465		
102	20/05/2015 22.48	-10.8633	163.997	10.2	6.8	Solomon Is. [Sea]	No Correlation	15456		

103	12/05/2015 21.13	38.8283	142.236	57.6	6.8	Near east coast of eastern Honshu, Japan [Sea: Japan]	No Correlation	9633		
104	01/05/2015 08.06	-5.13984	151.685	19.5	6.8	Papua New Guinea [Land]	No Correlation Papua New Guinea	14200		
105	30/04/2015 10.45	-5.29453	151.784	19.5	6.8	Papua New Guinea [Land]	No Correlation Papua New Guinea	14189		
106	13/02/2015 18.59	52.654	-31.998	16.8	6.8	Northern Mid-Atlantic Ridge [Sea]	No Correlation	3567		
107	09/10/2014 02.14	-31.704	-110.75	77.3	6.8	Easter Island region [Sea]	No Correlation	15024		
108	11/07/2014 19.22	37.069	142.364	13.3	6.8	Off east coast of Honshu, Japan [Sea: Japan]	No Correlation	9780		
109	15/12/2019 06.11	6.63398	125.321	19.9	6.7	Philippines [Land]	No Groundwater level data	11254		
110	12/04/2019 11.40	-1.80703	122.632	19.5	6.7	Sulawesi, Indonesia [Sea: Indonesia]	No Correlation	11300		
111	22/01/2019 19.01	-42.8695	42.2754	19.5	6.7	Prince Edward Islands, South Africa, region [Sea]	No Correlation	9845		
112	15/01/2019 18.06	-13.1977	166.721	19.5	6.7	Vanuatu Islands [Sea: Vanuatu]	No Correlation	15500		
113	22/10/2018 06.16	49.152	-129.391	9.8	6.7	Canada [Sea]	No Correlation	9272		
114	09/09/2018 19.31	-9.99844	161.423	98.4	6.7	Solomon Is. [Sea]	No Correlation	15445		
115	29/08/2018 03.51	-21.9727	170.087	19.5	6.7	New Caledonia [Sea]	No Correlation	16500		
116	24/08/2018 09.04	-10.9898	-70.6128	636.7	6.7	Peru [Land]	No Correlation depth>150km	10330		

117	21/08/2018 22.32	-16.05	168.15	30.3	6.7	Vanuatu Islands [Sea: Vanuatu]	No Correlation	16200		
118	26/03/2018 09.50	-5.42109	151.376	19.5	6.7	Papua New Guinea [Land]	No Correlation Papua New Guinea	14566		
119	08/03/2018 17.39	-4.42266	153.232	19.5	6.7	Papua New Guinea [Sea]	No Correlation Papua New Guinea	14500		
120	13/11/2017 02.28	9.56602	-84.5332	34.8	6.7	Costa Rica	No Correlation	9975		
121	04/11/2017 09.00	-15.293	-173.152	10.2	6.7	Tonga Islands [Sea: Tonga]	No Correlation	17400		
122	20/07/2017 22.31	36.9088	27.4504	10	6.7	Turkey [Sea]	Correlation	1312	1.8	15
123	29/05/2017 14.35	-1.25859	120.466	19.5	6.7	Sulawesi, Indonesia [Land: Indonesia]	No Correlation	11560		
124	24/04/2017 21.38	-32.9818	-71.9231	10	6.7	Chile (Peruvian point of view) [Sea]	No Correlation	11900		
125	29/03/2017 04.09	56.8758	162.669	18	6.7	Near east coast of Kamchatka Peninsula, Russia [Land: Russia]	No Correlation	8649		
126	21/12/2016 00.17	-7.47422	127.948	164.8	6.7	Indonesia [Sea]	No Correlation depth>150km	12545		
127	20/12/2016 04.21	-10.201	161.231	35	6.7	Solomon Is. [Sea]	No Correlation	15500		
128	28/05/2016 05.38	-21.8848	-178.101	408.2	6.7	Fiji Islands region [Sea: Fiji]	No Correlation depth>150km	16980		
129	06/04/2016 06.58	-14.0133	166.545	19.5	6.7	Vanuatu Islands [Sea: Vanuatu]	No Correlation	16000		
130	11/01/2016 16.38	3.86	127.03	18	6.7	Talaud Islands, Indonesia [Sea: Indonesia]	No Correlation	11500		
131	03/01/2016 23.05	24.743	93.6608	19.5	6.7	India [Land]	No Correlation	7397		

132	13/11/2015 20.51	30.91	128.79	10	6.7	Joint regime Japan/Korea [Sea]	No Correlation	9550		
133	11/11/2015 01.54	-29.4539	-71.7747	19.5	6.7	Chile (Peruvian point of view) [Sea]	No Correlation	11878		
134	13/09/2015 08.14	25.1191	-109.393	18.4	6.7	Gulf of California, Mexico [Sea: Mexico]	No Correlation	10568		
135	26/04/2015 07.09	27.6961	85.981	10.2	6.7	Nepal	No Correlation	6601		
136	27/02/2015 13.45	-7.25273	122.509	552.7	6.7	Indonesia [Sea]	No Correlation depth>150km	12085		
137	08/12/2014 08.54	8.05	-82.7	19.5	6.7	Panama [Sea]	No Correlation	9941		
138	26/11/2014 14.33	1.888	126.531	113	6.7	Indonesia [Sea]	No Correlation	11707		
139	16/11/2014 22.33	-37.842	179.759	35	6.7	Off east coast of North Island, New Zealand [Sea: New Zealand]	No Correlation	18730		
140	01/11/2014 18.57	-19.638	-177.761	408.6	6.7	Fiji Islands region [Sea: Fiji]	No Correlation depth>150km	17277		
141	17/09/2014 06.14	13.704	144.397	164.8	6.7	Northern Mariana Islands-Guam [Sea]	No Correlation depth>150km	12025		
142	29/09/2019 15.57	-35.4076	-73.1711	13.1	6.6	Chile (Peruvian point of view) [Sea]	No Correlation	12327		
143	27/08/2019 23.55	-60.0258	-26.6276	124.2	6.6	South Georgia & the South Sandwich Is. [Sea]	No Correlation	11919		
144	01/08/2019 18.28	-34.1666	-72.3757	19.7	6.6	Chile (Peruvian point of view) [Sea]	No Correlation	12181		
145	07/07/2019 15.08	0.485156	126.176	19.5	6.6	Indonesia [Sea]	No Correlation	11795		
146	22/01/2019 05.10	-10.3711	119.046	19.5	6.6	Indonesia [Sea]	No Correlation	12025		
147	20/01/2019 01.32	-30.1113	-71.5772	61.3	6.6	Chile (Peruvian point of view) [Sea]	No Correlation	11846		
148	05/01/2019 19.25	-8.09297	-71.6414	594.5	6.6	Western Brazil [Land: Brazil]	No Correlation depth>150km	10244		

149	18/11/2018 20.25	-17.9648	-178.777	536.7	6.6	Fiji Islands region [Sea: Fiji]	No Correlation depth>150km	17082		
150	22/10/2018 05.39	49.25	-129.47	11	6.6	Canada [Sea]	No Correlation	9258		
151	16/10/2018 00.28	-21.8039	169.507	10.2	6.6	New Caledonia [Sea]	No Correlation	16814		
152	10/10/2018 23.16	49.1273	156.193	10.2	6.6	Kuril Islands, Russia [Sea: Russia]	No Correlation	9245		
153	30/09/2018 10.52	-18.3164	-178.051	553.9	6.6	Fiji Islands region [Sea: Fiji]	No Correlation depth>150km	17035		
154	15/08/2018 21.56	51.3352	-177.99	19.5	6.6	Andreanof Islands, Aleutian Islands, United States [Sea: United States]	No Correlation	9582		
155	28/07/2018 22.47	-8.31797	116.543	19.5	6.6	Indonesia [Land]	No Correlation	11675		
156	04/05/2018 22.33	19.6523	-155.123	10.2	6.6	Hawaiian Islands, United States [Land: United States]	No Correlation	13065		
157	19/11/2017 15.09	-21.4664	168.635	19.5	6.6	New Caledonia [Sea]	No Correlation	16739		
158	31/10/2017 00.42	-21.6352	169.17	10.2	6.6	New Caledonia [Sea]	No Correlation	16776		
159	13/08/2017 03.08	-3.61055	101.732	64.5	6.6	Southern Sumatera, Indonesia [Sea: Indonesia]	No Correlation	10095		
160	09/05/2017 13.52	-14.5477	167.322	186.7	6.6	Vanuatu Islands [Sea: Vanuatu]	No Correlation depth>150km	16027		
161	03/04/2017 17.40	-22.5633	25.152	10.2	6.6	Botswana	No Correlation	7260		
162	19/01/2017 23.04	-10.3535	161.293	34.8	6.6	Solomon Is. [Sea]	No Correlation	15274		
163	24/09/2016 21.28	-19.7473	-178.162	598.8	6.6	Fiji Islands region [Sea: Fiji]	No Correlation depth>150km	17275		

164	31/08/2016 03.11	-3.67734	152.782	500.8	6.6	Papua New Guinea [Sea]	No Correlation Papua New Guinea	14125		
165	10/04/2016 10.28	36.3727	71.2002	196.1	6.6	Afghanistan [Land]	No Correlation depth>150km	4895		
166	08/02/2016 16.19	-6.58828	154.709	19.5	6.6	Papua New Guinea [Sea]	No Correlation Papua New Guinea	14503		
167	14/01/2016 03.25	41.915	142.786	56.8	6.6	Japan [Sea]	No Correlation	9385		
168	10/07/2015 04.12	-9.31641	158.309	10.2	6.6	Solomon Is. [Sea]	No Correlation	14995		
169	11/02/2015 18.57	-23.027	-66.636	188.3	6.6	Jujuy Province, Argentina [Land: Argentina]	No Correlation depth>150km	10957		
170	07/12/2014 01.22	-6.518	154.413	19.5	6.6	Papua New Guinea [Sea]	No Correlation Papua New Guinea	14478		
171	07/11/2014 03.33	-6.03	148.24	63	6.6	Papua New Guinea [Sea]	No Correlation Papua New Guinea	14003		
172	23/08/2014 22.32	-32.702	-71.387	32	6.6	Near coast of central Chile [Land: Chile]	No Correlation	12012		
173	04/07/2014 15.00	-6.205	152.821	10	6.6	Papua New Guinea [Sea]	No Correlation Papua New Guinea	14005		
174	04/11/2019 22.43	-18.457	-175.3	10.2	6.5	Tonga Islands [Sea: Tonga]	No Correlation	17236		
175	31/10/2019 01.11	6.93633	125.314	19.9	6.5	Philippines [Land]	No Correlation	11223		
176	29/10/2019 01.04	6.79922	125.205	19.5	6.5	Philippines [Land]	No Correlation	11225		
177	25/09/2019 23.46	-3.43828	128.44	19.5	6.5	Seram, Indonesia [Sea: Indonesia]	No Correlation	12279		

178	14/07/2019 05.39	-18.0527	120.33	9.8	6.5	Western Australia [Sea: Australia]	No Correlation	12688		
179	19/06/2019 07.01	-30.4383	-177.643	19.5	6.5	Kermadec Islands, New Zealand [Sea: New Zealand]	No Correlation	18352		
180	18/06/2019 13.22	38.5629	139.504	34.8	6.5	Near west coast of eastern Honshu, Japan [Sea: Japan]	No Correlation	9518		
181	16/06/2019 05.17	-31.2117	-177.691	10.2	6.5	New Zealand [Sea]	No Correlation	18418		
182	09/04/2019 17.54	-58.507	-25.1953	83.6	6.5	South Georgia & the South Sandwich Is. [Sea]	No Correlation	11733		
183	06/03/2019 15.46	-31.5562	-177.891	18.8	6.5	New Zealand [Sea]	No Correlation	18440		
184	01/02/2019 16.14	14.8887	-92.157	98	6.5	Guatemala [Land]	No Correlation	10180		
185	09/11/2018 01.49	71.5816	-11.3555	9.8	6.5	Jan Mayen Island region [Sea: Jan Mayen]	No Correlation	3555		
186	22/10/2018 06.22	49.314	-129.673	10	6.5	Canada [Sea]	No Correlation	9260		
187	13/10/2018 11.10	52.6992	153.314	458.6	6.5	Northwest of Kuril Islands, Russia [Sea: Russia]	No Correlation depth>150km	8790		
188	10/10/2018 22.00	-4.94297	151.713	139.8	6.5	Papua New Guinea [Land]	No Correlation Papua New Guinea	14168		
189	28/08/2018 22.35	16.6148	146.887	19.5	6.5	Northern Mariana Islands-Guam [Sea]	No Correlation	11915		
190	15/12/2017 16.48	-7.61836	108.144	129.3	6.5	Jawa, Indonesia [Land: Indonesia]	No Correlation	10931		
191	08/12/2017 00.22	10.006	140.252	19.5	6.5	Western Caroline Islands, Micronesia [Sea: Micronesia]	No Correlation	12062		

192	07/11/2017 21.26	-4.17305	143.525	112.1	6.5	New Guinea, Papua New Guinea [Land: Papua New Guinea]	No Correlation Papua New Guinea	13507		
193	01/11/2017 02.24	-21.5367	168.865	19.5	6.5	New Caledonia [Sea]	No Correlation	16750		
194	10/10/2017 18.53	-54.0352	8.26823	0.8	6.5	Bouvet I. [Sea]	No Correlation	10680		
195	31/08/2017 17.06	-1.14258	99.777	73.8	6.5	Southern Sumatera, Indonesia [Sea: Indonesia]	No Correlation	9766		
196	08/08/2017 23.27	44.2055	82.8418	10.2	6.5	Northern Xinjiang, China [Land: China]	No Correlation	5460		
197	02/06/2017 22.24	54.007	170.872	10.2	6.5	Near Islands, Aleutian Islands, United States [Sea: United States]	No Correlation	9120		
198	10/05/2017 23.23	-56.2992	-25.8724	19.5	6.5	South Georgia & the South Sandwich Is. [Sea]	No Correlation	11533		
199	07/02/2017 22.03	25.0945	63.2278	10.2	6.5	Southwestern Pakistan [Sea: Pakistan]	No Correlation	4875		
200	10/01/2017 15.27	-10.0758	161.009	19.5	6.5	Solomon Is. [Sea]	No Correlation	15236		
201	20/12/2016 04.21	-10.09	160.995	19.5	6.5	Solomon Is. [Sea]	No Correlation	15227		
202	20/11/2016 20.57	-31.4086	-68.4988	110.2	6.5	San Juan Province, Argentina [Land: Argentina]	No Correlation	11700		
203	30/10/2016 06.40	42.8303	13.1092	10	6.5	4 km NE Norcia (PG)	Correlation	93	1.2	5
204	01/06/2016 22.56	-2.01445	100.719	87.9	6.5	Southern Sumatera, Indonesia [Sea: Indonesia]	No Correlation	9897		
205	18/05/2016 07.57	0.457031	-79.7695	19.5	6.5	Near coast of Ecuador [Land: Ecuador]	No Correlation	10277		
206	05/02/2016 19.57	22.7461	120.808	19.5	6.5	Taiwan	No Correlation	9638		

207	17/12/2015 19.49	15.9082	-93.3142	109.8	6.5	Near coast of Chiapas, Mexico [Land: Mexico]	No Correlation	10184		
208	17/11/2015 07.10	38.7413	20.5236	10.1	6.5	Costa Greca Ionica (GRECIA)	Correlation	683	3.1	10
209	04/11/2015 03.44	-8.27051	124.951	10	6.5	Indonesia [Land]	No Correlation	12358		
210	15/08/2015 07.47	-10.8492	163.777	10.2	6.5	Solomon Is. [Sea]	No Correlation	15473		
211	10/08/2015 04.12	-9.28828	158.027	10.2	6.5	Solomon Is. [Sea]	No Correlation	14965		
212	20/06/2015 02.10	-36.2461	-73.4502	10.2	6.5	Chile (Peruvian point of view) [Sea]	No Correlation	12407		
213	20/04/2015 01.42	24.121	122.508	18.4	6.5	Japan [Sea]	No Correlation	9655		
214	20/02/2015 04.25	39.8514	143.562	10	6.5	Off east coast of Honshu, Japan [Sea: Japan]	No Correlation	9400		
215	16/02/2015 22.00	-55.315	-28.164	10.2	6.5	South Georgia & the South Sandwich Is. [Sea]	No Correlation	11520		
216	23/01/2015 03.47	-17.04	168.55	216	6.5	Vanuatu Islands [Sea: Vanuatu]	No Correlation depth>150km	16335		
217	21/11/2014 10.10	2.246	127.1	63.7	6.5	Indonesia [Sea]	No Correlation	11732		
218	20/07/2014 18.32	44.72	148.79	10	6.5	Conflict zone Japan/Russia [Sea]	No Correlation	9388		

Supplementary Table 2

Earthquake ID	Time (UTC)	Magnitude	EventLocationName	Detrended period	Mean value	±2σ	Max exceeding value
1	26/5/19 7.41	8.2	Northern Peru [Land: Peru]	15 days	0.004	0.016	-0.018
2	16/9/15 22.54	8.2	Near coast of central Chile [Land: Chile]	15 days	-0.009	0.009	-0.041
3	19/8/18 0.19	8.1	Fiji Islands region [Sea: Fiji]	3 days	0.011	0.008	0.020
5	13/11/16 11.03	8.1	South Island, New Zealand [Land: New Zealand]	8 days	-0.005	0.013	0.019
6	8/9/17 4.49	8	Near coast of Chiapas, Mexico [Sea: Mexico]	6 days	0.000	0.007	0.025
8	8/12/16 17.38	7.8	Solomon Is. [Sea]	5 days	-0.004	0.007	0.069
9	16/4/16 23.58	7.8	Near coast of Ecuador [Land: Ecuador]	5 days	0.001	0.003	0.007
11	25/4/15 6.11	7.7	Nepal	-	-	-	-
14	17/7/17 23.34	7.6	Russia [Sea]	7 days	-0.002	0.005	0.008
15	25/12/16 14.22	7.6	Southern Chile [Land: Chile]	10 days	-0.001	0.008	-0.024
17	2/3/16 12.49	7.6	Southwest of Sumatera, Indonesia [Sea]	-	-	-	-
28	19/8/16 7.32	7.3	South Georgia & the South Sandwich Is. [Sea]	-	-	-	-
48	20/12/18 17.01	7	Russia [Sea]	5 days	0.005	0.004	-0.014
54	15/4/16 16.25	7	Kyushu, Japan [Land: Japan]	5 days	0.001	0.003	0.010
73	7/12/15 7.50	6.9	Tajikistan	4 days	0.006	0.004	0.018

122	20/7/17<br>22.31	6.7	Turkey [Sea]	7 days	-0.002	0.005	0.008
203	30/10/16<br>6.40	6.5	4 km NE Norcia (PG)	-	-	-	-
208	17/11/15<br>7.10	6.5	Costa Greca Ionica (GRECIA)	5 days	-0.004	0.004	-0.023
